# Benefiting From Digital Use: Prospective Association of Internet Use With Knowledge and Preventive Behaviors Related to Alzheimer Disease in the Israeli Survey of Aging

**DOI:** 10.2196/25706

**Published:** 2021-04-30

**Authors:** Efrat Neter, Svetlana Chachashvili-Bolotin, Bracha Erlich, Kfir Ifrah

**Affiliations:** 1 Department Behavioral Sciences, Faculty of Social & Community Sciences Ruppin Academic Center Emeq Hefer Israel; 2 Institute for Immigration & Social Integration, Faculty of Social & Community Sciences Ruppin Academic Center Emeq Hefer Israel; 3 Braun School of Public Health and Community Medicine The Hebrew University of Jerusalem Jerusalem Israel; 4 Gerontological Data Center, The Paul Baerwald School of Social Work and Social Welfare The Hebrew University of Jerusalem Jerusalem Israel; 5 Gerontological Psychology, Faculty of Social & Community Sciences Ruppin Academic Center Emeq Hefer Israel

**Keywords:** Alzheimer disease, digital benefits, digital divide, digital skills, internet use, health behaviors, social capital

## Abstract

**Background:**

Previous work documented the beneficial association between internet use and improved cognition, functional capacity, and less cognitive decline among people in late adulthood. This work focused on potential mechanisms of such an association: knowledge on Alzheimer disease (AD) and preventive behaviors related to AD.

**Objective:**

The aim of this study was to examine prospective associations of internet use and perceived computer skills with knowledge on AD and preventive behaviors related to AD.

**Methods:**

The sample included 1232 older adults (mean age 71.12 [SD 9.07]) drawn from the Israeli branch of the Survey of Health, Aging, and Retirement in Europe (SHARE-Israel). The sample is representative of Israeli households of adults aged 50 or older and their spouses. Data analyzed were collected in person during 2015 (Wave 6), and in a drop-off questionnaire following the in-person 2017 data collection (Wave 7).

**Results:**

Although both internet use and perceived computer skills were prospectively associated with knowledge and behaviors related to AD in bivariate analyses, after controlling for sociodemographics, only internet use was associated with more such knowledge (*β*=.13, *P*<.001) and behaviors (*β*=.22, *P*<.001).

**Conclusions:**

Internet use emerged as a prospective predictor of protective factors against AD. Policymakers should advance digital engagement so as to enhance knowledge on AD and preventive behaviors among older adults.

## Introduction

### Background

Alzheimer disease (AD) is the most commonly occurring form of dementia, appearing predominantly in late adult life [1,2]. The disease causes a progressive cognitive, behavioral, and functional impairment, taking a heavy personal and financial toll on the patient, his/her family, and social services [3].

Although the neuropathological features resulting in neurodegeneration are well-recognized, the primary pathogenic factors and processes remain unclear. Hence, no effective treatments that can prevent the onset and progression of the disease exist hitherto, and much attention is directed at primary prevention [4-6]. The sporadic forms of AD (as opposed to familial), which comprise the majority of cases, have multifactorial etiology comprising both genetic and potentially modifiable risk factors. The identified risk factors include depression, diabetes, physical inactivity, (midlife) hypertension, (midlife) obesity, smoking, high cholesterol, coronary heart disease, renal dysfunction, and low unsaturated fat intake, whereas the protective factors include high cognitive activity, low/moderate alcohol consumption, and Mediterranean diet [7].

Some evidence—both reviews [5,8,9] and randomized controlled trials [10,11]—indicates that cognitive stimulation in various forms can delay cognitive decline. Digital engagement—engaging in computers and the internet—could be surmised to be a form of cognitive stimulation, carried out both at leisure and at work. Indeed, recent studies found that internet use was associated with a reduction in dementia, even after controlling for health status, marital status, and numeracy skills [12], and that internet use was associated with improved cognition, functional capacity, and quality of life [13-15]. However, the mechanisms through which internet use was associated with improved cognition were not explicated. In her review of internet use as a prevention tool against cognitive decline, spanning online cognitive training programs, internet conversation, and use of internet/email, Klimova [9] postulated 2 mechanisms through which this effect might take place: (1) simply acquiring health knowledge and (2) transforming knowledge into norms and behaviors [16-18]. Knowledge about a health condition is a critical first step in facilitating appropriate and timely use of preventive health services [19] and behaviors, which are documented as risk/protective factors to AD [7].

The benefits accrued through internet use resonate the discourse on digital divide, specifically the “third digital divide” [20-24]. The concept of “digital divide” evolved in the last 2 decades. The initial focus was on people’s physical/material access to computers, internet, and broadband internet, which was retrospectively labeled the “first digital divide.” As more people gained access to digital media in developed countries [25,26], the interest in digital inequality has shifted from physical availability to how people use the technology [22,27], labeled the “second digital divide” or “usage gap” [28]. As the skill level of many people increased, the interest in the digital divide shifted to gains/outcomes/impacts or benefits that ensue from internet usage, labeled the “third digital divide.” This third digital divide is postulated to correspond to offline economic, cultural, social, and personal resources/domains [29]. In the health domain, benefits from internet use span, to name a few, from comparing and switching health insurance, using high-reputed internet sources, arriving well-prepared to a medical visit to adopting positive health behaviors [29].

This study focused on potential pathways between internet use and improved cognition—knowledge and behavior—interpreting them as benefits of internet use. It examined the first phase of the link: the association between internet use and perceived computer skills, on the one hand, and knowledge on AD and AD-related preventive behaviors, on the other hand. Specifically, do people who are more engaged digitally know more about AD, and are they more engaged in modifiable behavioral risk/protective factors? The analysis was carried out on data from the Israeli branch of the Survey of Health, Aging, and Retirement in Europe (SHARE-Israel), allowing for a prospective examination between the focal variables and controlling for covariates, both demographic and medical risk factors for AD. The demographic variables controlled for were gender, age, income adequacy, and education, often controlled for in internet use studies [15,30]. The medical risk factors controlled for were chronic conditions, as they may be associated with AD-preventive behaviors or knowledge on AD. Protective behaviors related to AD were not controlled for, as they were one of the focal dependent variables.

### The Study Hypotheses

Based on the literature reviewed above, the study examines 2 hypotheses:

*Hypothesis 1a*: Internet use is prospectively associated with knowledge on AD and AD-related preventive behaviors.

*Hypothesis 1b*: Perceived computer skills are prospectively associated with knowledge on AD and AD-related preventive behaviors.

*Hypothesis 2a*: The above prospective associations between internet use and knowledge on AD and AD-related preventive behaviors hold also after controlling for background variables of gender, age, education, income adequacy, and reported medical risk factors.

*Hypothesis 2b*: The above prospective associations between perceived computer skills and knowledge on AD and AD-related preventive behaviors hold also after controlling for the aforementioned background variables.

## Methods

### Participants and Procedure

Data were drawn from 2 waves of the SHARE-Israel. The SHARE is a cross-national (27 European countries and Israel) panel database of microdata on health, socioeconomic status, and social and family networks [31], whose purpose is to provide a broad picture of life after the age of 50 for researchers and policy makers. Waves of data collection started in 2004, and thus far 7 waves were collected, the latest in 2017. The Israeli sample is representative of Israeli households of adults aged 50 or older and their spouses (the latter regardless of age) [32]. The design was based on a probability sample of households within 150 representative statistical areas delineated by geographical and sociodemographic criteria. More details on SHARE-Israel can be found on its official website [33].

In this study, respondents were interviewed during 2015 (Wave 6), when they were asked about internet use and perceived computer skills. They also responded to a supplementary paper drop-off questionnaire focusing on AD during 2017 (Wave 7). The data were collected by a comprehensive face-to-face interview using a computer-assisted personal interview, which lasted about 90 minutes, and a supplementary paper drop-off questionnaire, which was returned later. Informed consent had been obtained from all respondents prior to the interview. SHARE-Israel received ethical approval from the Institutional Review Board of the Hebrew University of Jerusalem and the general survey had a countries-wide institutional review board [34].

As the survey questions regarding AD were included in the drop-off questionnaire administered at Wave 7, the sample of this study was limited to the 1232 respondents who completed this questionnaire and also participated in Wave 6 where they were asked on internet use and perceived computer skills. Response to the surveys was either in Hebrew, Arabic, or Russian, the 3 major languages spoken in Israel among adults aged 50 and over (Table 1).

**Table 1:** Descriptive characteristics of the study sample.

Variable				Value
Age (years), mean (SD); range				71.12 (9.07); 42-101
**Gender (n=1232), n (%)**				
	0=men			516 (41.88)
	1=women			716 (58.12)
**Cohabitation (n=1232), n (%)**				
	0=Living without a partner			330 (26.79)
	1=Living with a partner			902 (73.21)
**Education (n=1222), n (%)**				
	0=Never attended an education program			56 (4.58)
	1=Primary education			243 (19.89)
	2=Lower secondary education			126 (10.31)
	3=Upper secondary education			307 (25.12)
	4=Postsecondary nontertiary education			73 (5.97)
	5=Bachelor’s or equivalent level			412 (33.72)
	6=Master’s or Doctoral equivalent level			5 (0.41)
**Perceived income adequacy (n=1214), n (%)**				
	1=With great difficulty			126 (10.38)
	2=With some difficulty			307 (25.29)
	3=Fairly easily			345 (28.42)
	4=Easily			436 (35.91)
**Medical risk factors (n=1217), mean (SD); range**				1.46 (1.40); 0-7
	**Internet use (during past 7 days) (n=1232), n (%)**			
		0=No	590 (47.89)	
		1=Yes	642 (52.11)	
	**Perceived computer skills (n=1229), n (%)**			
		0=I never used a computer	350 (28.48)	
		1=Poor	97 (7.89)	
		2=Fair	229 (18.63)	
		3=Good	262 (21.32)	
		4=Very good	188 (15.30)	
		5=Excellent	95 (7.73)	
	**Knowledge on** **Alzheimer** **disease**			
		Total score (n=1232), mean (SD); range	4.68 (2.31); 0-10	
	**Alzheimer disease–related preventive behaviors**			
		Total score (n=1232), mean (SD); range	3.75 (2.09); 0-9	

### Measures

#### Internet Use

Participants were asked whether they had used the internet in the past week for either email, information search, shopping, or any other purpose at least once. Responses were on a dichotomous yes/no scale (coded as “1” [Yes] or “0” [No]). This measure has been used in previous research to indicate general and regular internet usage behavior [15,35].

#### Perceived Computer Skills

Participants were asked, “How would you rate your computer skill? Would you say they are ...” A 5-point scale was used, ranging from *poor* to *excellent*. An additional category was *I never used a computer*. Responses were coded so that *I never used a computer* was the lowest end (0) of the scale and *excellent* was the highest (5). This measure has been used in previous research to indicate subjective skills [36,37].

#### Knowledge on Alzheimer Disease

Questions tapping general knowledge on AD were adapted [19,38]. All 10 items were pretested in 2 prior Israeli samples. Responses were provided on a 3-point response scale, including *correct/incorrect*/*I don’t know*. Sample items were “Alzheimer’s disease could be contagious” (false, [19]), “Alzheimer’s disease can be diagnosed with a blood test” (false, [19]), and “Symptoms of severe depression can be mistaken for symptoms of AD” (true, [38]) [see Multimedia Appendix 1]. Correct responses were summed up so that the knowledge on AD score ranged from 0 to 10. Cronbach α coefficient of the scale was fair, α=.63, considering it was based on dichotomous items [39].

#### Alzheimer Disease–Related Preventive Behaviors

Participants were asked to check whether they had engaged in the following behaviors on a regular basis in the past month. Nine behaviors were included: physical exercise; consumption of foods with high saturated fat and cholesterol; consumption of green vegetables, nuts, cereals, fish, or olive oil; limitation of total daily caloric intake; reduction of stress level, done in order to reduce, prevent, and cope with stress; communication with family or friends; participation in social activities; doing number games such as crossword puzzles or sudoku; and taking nutritional supplements, such as vitamin B_12_, vitamin E, and omega 3 fatty acids. The items were identified as AD-protective factors [7]. All items were pretested in 2 small prior samples. The items were summed up so that the score could potentially range from 0 to 9. Cronbach α coefficient of the scale was fair, α=0.62, considering it was based on dichotomous items [39].

Sociodemographic characteristics included gender (1=female; 0=male), age in years, education level, and perceived household income adequacy. Perceived income adequacy was assessed by asking, “Thinking of your household’s total monthly income, would you say that your household is able to make ends meet?.” The answer scale was composed of 4 categories ranging from 1=*with great difficulty* to 4=*easily* [40]*.* Education level was coded by 1 of 7 categories classified by the International Standard Classification of Education (ISCED), ranging from 0 (preprimary) to 6 (second-stage tertiary education) [41]. Medical risk factors, as based on the Wave 6 report, included coronary heart disease, hypertension, high cholesterol, diabetes, renal dysfunction, affective disorder, and BMI of 30 kg/m^2^ or above. These were summed up to form a *medical risk factors* score [42] ranging from 0 to 7.

### Data Analysis

We first performed univariate descriptive analyses, characterizing the study’s participants. Then, Pearson correlations and unpaired *t* tests were conducted in order to examine Hypothesis 1 on bivariate associations between the main variables (for continuous and categorical variables, respectively). We then tested Hypothesis 2 in a series of hierarchical multiple regression analyses examining internet use and perceived computer skills’ prediction of each of the dependent variables: knowledge on AD and AD-related preventive behaviors. As the predictors internet use and perceived computer skills were highly associated, they were not entered simultaneously, and thus separate regression analyses examined the prediction of each of these predictors. Step 1 in the regression included gender, age, education, and income adequacy, demographic variables traditionally controlled for in studying internet use [15]. We also included reported medical risk factors in this step to control for the possibility that reported conditions could be associated with increased knowledge and preventive behaviors. Step 2 additionally included either internet use or perceived computer skills. Data were analyzed using SPSS statistical software, PC version 25.0 (IBM).

## Results

### Descriptive Characteristics of the Sample

Wave 7 included 2131 individuals; 1638 of them returned the AD drop-off questionnaire, and 1232 of them also had data from the Wave 6 data collection. A comparison of the larger group of all Wave 7 respondents with those included in this analysis yielded no significant differences (*P*=.30, .70, and .15 for age, gender, and education, respectively) except in income adequacy (*P*=.008), which was slightly higher (mean difference of 0.1) in our analysis sample. Characteristics of the participants are presented in Table 1. The mean age in the current sample at Wave 7 (n=1232) was 71.12 (SD 9.07). Most participants were women (716/1232, 58.12%) or living with a partner (902/1232, 73.21%). About one-third of the sample had lower secondary education or less (425/1222, 34.78%), a quarter (307/1232, 24.9%) had an upper secondary education, and 40.10% (490/1222) a postsecondary or tertiary education. As for the perceived income adequacy, most of the sample reported being able to make ends meet easily or fairly easily (781/1214, 64.33%). The medical risk factor mean was 1.46 (SD 1.40).

Regarding digital engagement, about one-half of the sample (642/1232, 52.11%) had used the internet during the 7 days prior to the interview. Almost one-half of the sample (545/1229, 44.34%) rated their computer skills as good to excellent. Knowledge on AD and AD-related preventive behaviors are also displayed in Table 1. The mean knowledge on AD was at midrange (mean 4.68, range 0-10), whereas the mean reported AD-related preventive behaviors was relatively low (mean 3.75, range 0-9).

### Bivariate Analyses

As displayed in Table 2, significant differences were found between respondents who had used the internet during the past week and those who had not in both knowledge on AD (t_1230_=7.33, *P*<.001, Cohen *d*=0.43) and AD-related preventive behaviors (t_1230_=9.08, *P*<.001, Cohen *d*=0.52), such that internet users reported higher knowledge on AD and AD-related preventive behaviors.

As presented in Table 3, the prospective correlations between perceived computer skills and both knowledge on AD and AD-related preventive behaviors were significant and positive, though weak (*r*=0.08, *P*<.001 and *r*=0.13, *P*<.001, respectively). The findings therefore provided support for Hypothesis 1. In addition, the correlations of education with internet use and perceived computer skills were significant and positive (*r*=0.46, *P*<.001 and *r*=0.43, *P*<.001, respectively), whereas the associations of age with internet use and perceived computer skills were significant and negative (*r*=–0.22, *P*<.001 and *r*=–0.32, *P*<.001, respectively).

**Table 2:** Knowledge on Alzheimer disease and Alzheimer disease–related preventive behaviors by internet use.

Pathway	Internet users (n=642), mean (SD)	Noninternet users (n=590), mean (SD)	*t* value (*df*)	*P*-value
Knowledge on Alzheimer disease	5.13 (2.33)	4.18 (2.20)	7.33 (1230)	<.001
Alzheimer disease–related preventive behaviors	4.25 (2.07)	3.20 (1.97)	9.08 (1230)	<.001

**Table 3:** Correlations between the study variables.^a^

Study variables	KAD^b^	ADPB^c^	Internet use	Perceived computer skills	Education	Age
KAD	1					
ADPB	0.42^d^	1				
Internet use	0.21^d^	0.25^d^	1			
Perceived computer skills	0.08^e^	0.13^d^	0.68^d^	1		
Education	0.23^d^	0.29^d^	0.46^d^	0.43^d^	1	
Age	–0.01	–0.01	–0.22^d^	–0.32^d^	–0.12^d^	1

^a^N varies from 1205 to 1232.

^b^KAD: Knowledge on Alzheimer disease.

^c^ADPB: Alzheimer disease–related preventive behaviors.

^d^*P*<.001.

^e^*P*=.008.

### Multivariate Analyses Predicting Knowledge on Alzheimer Disease

Table 4 presents the linear hierarchical regression models predicting knowledge on AD. Model 1 includes the demographic and medical risk factor variables. It shows a positive association of education and knowledge with AD: the higher the level of education, the better the knowledge on AD. A similar positive association of income adequacy (*P*=.006) and medical risk factors (*P*=.005) with knowledge on AD was found. No significant associations of gender (*P*=.87) and age (*P*=.84) with knowledge on AD were found.

To examine Hypothesis 2 regarding the positive association between internet use and knowledge on AD, we added internet use to our model (see Model 2a). There were 2 main findings. First, the prospective association of internet use with knowledge on AD was significant and positive (*P*<.001). Respondents who used internet revealed higher knowledge on AD compared with those who did not report this use. Therefore, this finding provided support for Hypothesis 2 regarding internet use. In addition, the adjusted *R*^2^ (final step) in the sample was 6.9%.

To examine Hypothesis 2 regarding the positive association between perceived computer skills and knowledge on AD, we added perceived computer skills to our model (see Model 2b). The association between perceived computer skills and knowledge on AD was not significant (*P*=.09). Thus, this finding did not provide support for Hypothesis 2b regarding perceived computer skills. Note that when controlling for perceived computer skills, medical risk factors emerged as a significant predictor of knowledge on AD (*P*=.002), such that the more medical risk factors, the higher the knowledge on AD.

**Table 4:** Hierarchical linear regression predicting knowledge on Alzheimer disease.

Predictors	Model 1 (n=1190)				Model 2a (n=1190)				Model 2b (n=1187)		
	B^a^	SE B^b^	*β* ^c^	B		SE B	*β*	B		SE B	*β*
Constant	3.11	0.58	—^d^	2.79		0.58	—	3.38		0.63	—
Gender (women=1)	0.02	0.13	.04	0.05		0.13	.01	0.00		0.13	.00
Age	0.00	0.01	.01	0.00		0.00	.01	–0.00		0.01	–.02
Education	0.30	0.04	.21^e^	0.23		0.04	.16^e^	0.31		0.04	.22^e^
Income adequacy	0.19	0.07	.08^f^	0.12		0.07	.05	0.21		0.07	.09^g^
Medical risk factors	0.14	0.05	.08	0.15		0.05	.09	0.13		0.05	.08^h^
Internet use	—	—	—	0.60		0.15	.13^d^	—		—	—
Perceived computer skills	—	—	—	—		—	—	–0.06		0.05	–0.04
Adjusted *R*^2^	0.057	—	—		0.069	—	—		0.058	—	—
*R*^2^ change	—	—	—		0.012	—	—		0.001	—	—

^a^Unstandardized beta coefficient.

^b^Standard error for the unstandardized beta (B).

^c^Standardized beta coefficient.

^d^—: Not available

^e^*P*<.001.

^f^*P*=.006

^g^*P*=.003.

^h^*P*=.002.

### Multivariate Analyses Predicting Alzheimer Disease–Related Preventive Behaviors

Table 5 presents the hierarchical linear regression model predicting AD-related preventive behaviors. Similar to our findings regarding knowledge on AD, a positive significant association between education and income with AD-related preventive behaviors was recorded (<.001 and .036, respectively), whereas no significant associations were found for the association of gender (*P*=.07), age (*P*=.78), and medical risk factors (*P*=.60) with AD-related preventive behaviors.

To examine Hypothesis 2 regarding the positive association between internet use and AD-related preventive behaviors, we added internet use to our model (see Model 2a). The prospective association of internet use was significant and positive (*P*<.001). Respondents who used the internet had higher AD-related preventive behaviors compared with those who did not report this use. Therefore, this finding provided support for Hypothesis 2a regarding internet use. However, when controlling for internet use, gender emerged as a significant predictor of AD-related preventive behaviors such that women had higher AD-related preventive behaviors than men (*P*=.03). In addition, the adjusted *R*^2^ (final step) in the sample was 7.7%.

To examine Hypothesis 2b regarding the positive association between perceived computer skills and AD-related preventive behaviors, we added perceived computer skills to our model (see Model 2b). The effect of perceived computer skills was positive yet insignificant (*P*=.09); indeed, the adjusted *R*^2^ in the sample hardly changed compared with Model 1 and was only 4.5% (*P*=.09). Thus, this finding did not provide support for Hypothesis 2b regarding perceived computer skills.

**Table 5:** Hierarchical linear regression predicting Alzheimer disease–related preventive behaviors.

Predictors	Model 1 (n=1190)				Model 2a (n=1190)				Model 2b (n=1187)		
	B^a^	SE B^b^	*β* ^c^	B		SE B	*β*	B		SE B	*β*
Constant	2.35	0.53	—^d^	2.14		0.53	—	1.96		0.57	—
Gender (women=1)	0.21	0.12	.05	0.26		0.12	.06^e^	0.23		0.12	.05
Age	–0.00	0.00	–.01	0.01		0.01	.03	0.01		0.01	.03
Education	0.23	0.04	.18^f^	0.13		0.04	.10^f^	0.21		0.04	.17^f^
Income adequacy	0.13	0.06	.06^g^	0.03		0.06	.01	0.09		0.07	.05
Medical risk factors	–0.03	0.05	–.02	–0.05		0.05	–.00	–0.02		0.05	–.02
Internet use	—	—	—	0.93		0.14	.22^f^	—		—	—
Perceived computer skills	—	—	—	—		—	—	0.07		0.04	.06
Adjusted *R*^2^	0.043	—	—		0.077	—	—		0.045	—	—
*R*^2^ change	—	—	—		.034	—	—		.002	—	—

^a^Unstandardized beta coefficient.

^b^Standard error for the unstandardized beta (B).

^c^Standardized beta coefficient.

^d^—: Not available

^e^*P*<.32.

^f^*P*<.001.

^g^*P*=.04.

## Discussion

### Principal Findings

Our findings indicate that internet use was significantly and positively associated with both knowledge on AD and AD-related preventive behaviors (Hypothesis 1). The prospective association between internet use and knowledge on AD held also when demographic attributes were controlled for (Hypothesis 2a). It is noteworthy that even though education and internet use were positively associated (*r*=0.46, *P*<.001), internet use still added to the explained variance in both knowledge on AD and AD-related preventive behaviors, after controlling for education and other demographic and medical risk factors (Hypothesis 2a), and internet use was a stronger predictor of knowledge on AD and AD-related preventive behaviors compared with education. Overall, the explained variance in knowledge on AD and AD-related preventive behaviors was significant yet relatively small (6.9% and 7.7%, respectively). Perceived computer skills were also significantly associated with both knowledge on AD and AD-related preventive behaviors (Hypothesis 1b). However, this bivariate prospective association did not hold in a multivariate analysis, when gender, age, education, perceived income adequacy, and medical risk factors were controlled for in predicting knowledge on AD and AD-related preventive behaviors (Hypothesis 2b). Overall, the explained variance was significant yet relatively small (6.9% and 7.7%, respectively).

### Comparison With Prior Work

Our findings exemplify the capital-enhancing effect of using the internet in 2 domains: information acquisition and making more “responsible” choices [43,44]; these were both documented in the context of AD, relevant to people in middle and late adulthood. The findings indicate that internet users build personal capital and resources in the form of knowledge and behaviors. Our findings not only record first divide (ie, access) and second divide (ie, skills) variables’ prospective association with a third divide (ie, outcomes), but also lend support to the sequentiality in the digital divide [45,46], namely, that access and skills in the digital arena (ie, digital access and skills reported in Wave 6 data collection) subsequently relate to offline outcomes in *another* domain (ie, knowledge and behavior in the health arena reported in Wave 7). The size of the explained variance in knowledge on AD and AD-related preventive behaviors attests to a relatively small effect of accrued capital gained from internet use, yet one similar to the small effects of internet use on other health processes [47] and of social capital on health, as documented in a recent meta-analysis [48].

Education was found to be a significant predictor of knowledge on AD and AD-related preventive behaviors. These findings echo recommendations regarding dementia prevention [2]; education, along with cognitive stimulation, is associated with building a cognitive reserve earlier in life. This reserve translates also into actual participation in health behaviors [49].

The study has several implications. First, by suggesting and testing mechanisms through which internet use may be associated with reduced dementia prevalence [12,14], namely, knowledge acquisition and preventive behavior, it signposts digital engagement as an intervention venue, recommended also by other researchers [44]. The intervention does not need to be directive and focused on AD, as it seems that people arrive to this domain on their own.

Although it is unclear which specific digital activities are associated with enhanced knowledge on AD and AD-related preventive behaviors, opening the digital realm to more older adults is promising, and integrates into the evidence on the beneficial effects of cognitive stimulation. The scope of potential intervention focused on enhanced digital engagement/literacy is big, as only about one-half (642/1232, 52.11%, similar to SHARE respondents’ average use [35]) of the respondents to this survey, a representative sample of households in this age bracket, had been digitally engaged in the preceding week, and almost one-third reported they never used a computer. Although many respondents own mobile phones, most often smartphones [50], they often do not realize its affordances. Internet use among this age group in Israel has risen in the past decade [30], but there is ample room for an additional increase.

### Strengths and Limitations

This study has a number of strengths. First, the prospective association between internet use, knowledge on AD, and AD-related preventive behaviors is novel, and attests to the benefits accrued by internet use. It is one of the manifestations of the third digital divide in the health domain. Second, the longitudinal design of the study, establishing temporality, suggests directionality of the relationship between the variables. Internet use and perceived computer skills had both been measured in a previous data collection wave, and they predicted knowledge on AD and AD-related preventive behaviors. Most studies on internet use are not longitudinal, and do not afford drawing such a conclusion. A third major strength of the study is the representativeness of the sample, consisting of a relatively large number of respondents (n=1232).

However, certain limitations of this study should also be noted. Foremost, all the variables were self-reported and originated from a single source—the respondent. Some of the predictors are essentially reliable and valid (eg, demographic attributes), some are reported practices (eg, digital engagement) that are more prone to self-presentation bias, and some are inherently perceptions (perceived computer skills). The latter are moderately (though significantly) associated with actual performance, as can be inferred from reported and performed eHealth literacy [51]. This bias in perceived computer skills may explain the finding that it was not associated with knowledge nor behavior in multivariate analyses: it may not have measured computer skills accurately enough. Second, we cannot be completely confident in the directionality of the relationship between internet use, knowledge on AD, and AD-related preventive behaviors; the longitudinal measurement of these variables does not guarantee that this was the actual sequence between them. Third, the 2 dependent variables exhibited relatively low reliability, probably as they employed dichotomous responses and covered different domains (eg, behaviors pertaining to diet, exercise, and social interaction). Lastly, the study did not examine the association of knowledge on AD and AD-related preventive behaviors with cognitive decline (though the latter was examined by other studies), nor whether knowledge on AD and AD-related preventive behaviors mediate the association between internet use and cognitive decline. Despite the limitations, the study is unique in that it documented—in a longitudinal design—how using the internet and having digital skills can generate positive outcomes for people in middle and late adulthood. The positive outcomes examined were knowledge on AD and behaviors which can modify the timing of AD, and the prospective design of the study allowed inferring the direction of the association: from internet and computer use to tangible benefits.

### Future Directions

Future work could focus on unravelling what exactly in internet use afforded the reported benefits. This study did not track people’s actual use of the internet but rather relied on their report of using it during the previous week. A more nuanced measurement of internet use (both eHealth and mHealth [mobile health]) could provide more precise information. For example, are the benefits derived from using knowledge and experiential accounts created by peers, such as in online health communities [16], aligned with Web 2.0 activities, or alternatively are the tangible benefits that result in obtaining information from traditional sources, such as hospitals, health maintenance organizations, and patients’ associations, aligned with Web 1.0 consumption [52]? Adopting a functional approach [53], it is of special interest to unravel which digital activities are associated with AD-related preventive behaviors. Another intriguing direction is to examine subpopulations such as immigrants or minorities; knowledge on AD and AD-related preventive behaviors of subpopulations are of interest as well as the facilitating effect of internet use. Lastly, future research could focus on cognitive decline as the dependent variable, and test whether knowledge on AD and AD-related preventive behaviors indeed function as mediators between internet use and reduced dementia, as documented in recent findings [12,14,15]. Concomitantly, the mechanism through which internet use is associated with preventive behaviors could be further explored to examine whether knowledge mediates this association. Future research should avoid cross-sectional design, and hold onto the longitudinal design, to allow inferring the direction of the association.

## Appendix

Multimedia Appendix 1 Knowledge on Alzheimer disease.

## Abbreviations

AD: Alzheimer disease
SHARE: Survey of Health, Aging, and Retirement in Europe

## References

[1] Barnes DE, Yaffe K (2011). The projected effect of risk factor reduction on Alzheimer's disease prevalence. Lancet Neurol.

[2] Livingston G, Sommerlad A, Orgeta V, Costafreda SG, Huntley J, Ames D, Ballard C, Banerjee S, Burns A, Cohen-Mansfield J, Cooper C, Fox N, Gitlin LN, Howard R, Kales HC, Larson EB, Ritchie K, Rockwood K, Sampson EL, Samus Q, Schneider LS, Selbæk Geir, Teri L, Mukadam N (2017). Dementia prevention, intervention, and care. Lancet.

[3] Prince M, Comas-Herrera A, Knapp M, Guerchet M, Karagiannidou M (2016). World Alzheimer report 2016: improving healthcare for people living with dementia: coverage, quality and costs now and in the future.

[4] Schiepers OJG, Köhler Sebastian, Deckers K, Irving K, O'Donnell CA, van den Akker M, Verhey FRJ, Vos SJB, de Vugt ME, van Boxtel MPJ (2018). Lifestyle for Brain Health (LIBRA): a new model for dementia prevention. Int J Geriatr Psychiatry.

[5] Di Marco LY, Marzo A, Muñoz-Ruiz Miguel, Ikram MA, Kivipelto M, Ruefenacht D, Venneri A, Soininen H, Wanke I, Ventikos Yiannis A, Frangi AF (2014). Modifiable lifestyle factors in dementia: a systematic review of longitudinal observational cohort studies. J Alzheimers Dis.

[6] Norton S, Matthews FE, Barnes DE, Yaffe K, Brayne C (2014). Potential for primary prevention of Alzheimer's disease: an analysis of population-based data. Lancet Neurol.

[7] Deckers K, van Boxtel MPJ, Schiepers OJG, de Vugt M, Muñoz Sánchez Juan Luis, Anstey KJ, Brayne C, Dartigues J, Engedal K, Kivipelto M, Ritchie K, Starr JM, Yaffe K, Irving K, Verhey FRJ, Köhler Sebastian (2015). Target risk factors for dementia prevention: a systematic review and Delphi consensus study on the evidence from observational studies. Int J Geriatr Psychiatry.

[8] Alves J, Magalhães R, Thomas RE, Gonçalves OF, Petrosyan A, Sampaio A (2013). Is There Evidence for Cognitive Intervention in Alzheimer Disease? A Systematic Review of Efficacy, Feasibility, and Cost-Effectiveness. Alzheimer Disease & Associated Disorders.

[9] Klimova B (2016). Use of the Internet as a prevention tool against cognitive decline in normal aging. Clin Interv Aging.

[10] Richard E, Jongstra S, Soininen H, Brayne C, Moll van Charante EP, Meiller Y, van der Groep B, Beishuizen CRL, Mangialasche F, Barbera M, Ngandu T, Coley N, Guillemont J, Savy S, Dijkgraaf MGW, Peters RJG, van Gool WA, Kivipelto M, Andrieu S (2016). Healthy Ageing Through Internet Counselling in the Elderly: the HATICE randomised controlled trial for the prevention of cardiovascular disease and cognitive impairment. BMJ Open.

[11] Richard E, Moll van Charante EP, Hoevenaar-Blom MP, Coley N, Barbera M, van der Groep A, Meiller Y, Mangialasche F, Beishuizen CB, Jongstra S, van Middelaar T, Van Wanrooij LL, Ngandu T, Guillemont J, Andrieu S, Brayne C, Kivipelto M, Soininen H, Van Gool WA (2019). Healthy ageing through internet counselling in the elderly (HATICE): a multinational, randomised controlled trial. The Lancet Digital Health.

[12] Almeida OP, Yeap BB, Alfonso H, Hankey GJ, Flicker L, Norman PE (2012). Older men who use computers have lower risk of dementia. PLoS One.

[13] d'Orsi E, Xavier AJ, Steptoe A, de Oliveira C, Ramos LR, Orrell M, Demakakos P, Marmot MG (2014). Socioeconomic and lifestyle factors related to instrumental activity of daily living dynamics: results from the English Longitudinal Study of Ageing. J Am Geriatr Soc.

[14] d'Orsi E, Xavier AJ, Rafnsson SB, Steptoe A, Hogervorst E, Orrell M (2018). Is use of the internet in midlife associated with lower dementia incidence? Results from the English Longitudinal Study of Ageing. Aging Ment Health.

[15] Kamin ST, Lang FR (2020). Internet Use and Cognitive Functioning in Late Adulthood: Longitudinal Findings from the Survey of Health, Ageing and Retirement in Europe (SHARE). J Gerontol B Psychol Sci Soc Sci.

[16] Zhao J, Ha S, Widdows R (2015). The influence of social capital on knowledge creation in online health communities. Inf Technol Manag.

[17] Nahapiet J, Ghoshal S (1998). Social Capital, Intellectual Capital, and the Organizational Advantage. The Academy of Management Review.

[18] Rodgers J, Valuev AV, Hswen W, Subramanian SV (2019). Social capital and physical health: An updated review of the literature for 2007-2018. Soc Sci Med.

[19] Ayalon L, Areán Patricia A (2004). Knowledge of Alzheimer's disease in four ethnic groups of older adults. Int J Geriatr Psychiatry.

[20] Bilbao-Osorio B, Crotti R, Dutta S, Lanvin B (2014). The Networked Readiness Index 2014: Benchmarking ICT Uptake in a World of Big Data. The Global Information Technology Report 2014.

[21] van Deursen AJ, Helsper EJ (2015). A nuanced understanding of Internet use and non-use among the elderly. European Journal of Communication.

[22] Warschauer M, Matuchniak T (2010). New Technology and Digital Worlds: Analyzing Evidence of Equity in Access, Use, and Outcomes. Review of Research in Education.

[23] Cotten SR, Ford G, Ford S, Hale TM (2014). Internet use and depression among retired older adults in the United States: a longitudinal analysis. J Gerontol B Psychol Sci Soc Sci.

[24] Scheerder AJ, van Deursen AJAM, van Dijk JAGM (2020). Taking advantage of the Internet: A qualitative analysis to explain why educational background is decisive in gaining positive outcomes. Poetics.

[25] Fox S, Duggan M (2013). Health online 2013.

[26] European Commission (2014). Eurobarometer: European citizens’ digital health literacy.

[27] Hargittai E, Piper AM, Morris MR (2018). From internet access to internet skills: digital inequality among older adults. Univ Access Inf Soc.

[28] van Deursen AJAM, van Dijk JAGM (2015). Toward a multifaceted model of Internet access for understanding digital divides: An empirical investigation. The Information Society.

[29] Helsper EJ (2012). A Corresponding Fields Model for the Links Between Social and Digital Exclusion. Commun Theor.

[30] Lissitsa S, Chachashvili-Bolotin S (2015). Does the wind of change blow in late adulthood? Adoption of ICT by senior citizens during the past decade. Poetics.

[31] Börsch-Supan Axel, Brandt M, Hunkler C, Kneip T, Korbmacher J, Malter F, Schaan B, Stuck S, Zuber S, SHARE Central Coordination Team (2013). Data Resource Profile: the Survey of Health, Ageing and Retirement in Europe (SHARE). Int J Epidemiol.

[32] Börsch-Supan A (2020). Survey of Health, Ageing and Retirement in Europe (SHARE) Wave 6-7. Release version: 7.0.0. SHARE-ERIC. Data set.

[33] Survey of Health, Ageing and Retirement in Israel.

[34] Wolfrum R (2018). Opinion of the Ethics Council of the Max Planck Society on the SHARE-Project.

[35] König R, Seifert A, Doh M (2018). Internet use among older Europeans: an analysis based on SHARE data. Univ Access Inf Soc.

[36] Midão L, Pedreiro E, Pinho M, Dias I, Almada M, Garcia K, Rodrigues L, Christensen C, Pereira P, Bertram M, Busse G, Quarta B, Poulain M, Heery D, Ruseva G, Irbe M, Amaral M, Costa E (2020). Computer Skills Among the Community-Dwelling 55+ European Population Based on Survey of Health, Ageing, and Retirement in Europe. Int J Digit Lit Digit Competence.

[37] Reed K, Doty HD, May DR (2005). The impact of aging on self-efficacy and computer skill acquisition. J Manag Issues.

[38] Carpenter BD, Balsis S, Otilingam PG, Hanson PK, Gatz M (2009). The Alzheimer's Disease Knowledge Scale: development and psychometric properties. Gerontologist.

[39] Cohen J (2016). The Cost of Dichotomization. Applied Psychological Measurement.

[40] Litwin H, Sapir EV (2009). Perceived income adequacy among older adults in 12 countries: findings from the survey of health, ageing, and retirement in Europe. Gerontologist.

[41] Hoffmeyer-Zlotnik J.H.P., Wolf C, United Nations Educational, Scientific and Cultural Organization (UNESCO) (2003). International Standard Classification of Education, ISCED 1997. Advances in Cross-National Comparison.

[42] Luchsinger JA, Mayeux R (2004). Cardiovascular risk factors and Alzheimer's disease. Curr Atheroscler Rep.

[43] Folland S (2008). An economic model of social capital and health. Health Econ Policy Law.

[44] Choi M (2020). Association of eHealth Use, Literacy, Informational Social Support, and Health-Promoting Behaviors: Mediation of Health Self-Efficacy. Int J Environ Res Public Health.

[45] Van Deursen AJAM, Helsper E, Eynon R, van Dijk JAGM (2017). The compoundness and sequentiality of digital inequality. Int J of Communication.

[46] Lissitsa S, Chachashvili-Bolotin S, Bokek-Cohen Y (2017). Digital skills and extrinsic rewards in late career. Technology in Society.

[47] Neter E, Brainin E, Baron-Epel O, Hale T, Chou VS, Cotten S (2018). The Third Digital Divide in the Health Domain: Is Internet Use for Health Purposes Associated with Health Benefits?. eHealth: Current Evidence, Promises, Perils and Future Directions (Studies in Media and Communications, Vol. 15).

[48] Xue X, Reed WR, Menclova A (2020). Social capital and health: a meta-analysis. J Health Econ.

[49] Hagger M, Hamilton K (2021). Effects of socio-structural variables in the theory of planned behavior: a mediation model in multiple samples and behaviors. Psychol Health.

[50] Israel Bureau of Statistics (2017). Annual social survey; Expanded domain: health and lifestyle. https://www.cbs.gov.il/he/publications/doclib/2019/seker_hevrati17_1761/intro_h.pdf.

[51] Neter E, Brainin E (2017). Perceived and Performed eHealth Literacy: Survey and Simulated Performance Test. JMIR Hum Factors.

[52] Schradie J (2011). The digital production gap: The digital divide and Web 2.0 collide. Poetics.

[53] Lifshitz R, Nimrod G, Bachner YG (2018). Internet use and well-being in later life: a functional approach. Aging Ment Health.

