# Supplementary material for: Benefiting From Digital Use: Prospective Association of Internet Use With Knowledge and Preventive Behaviors Related to Alzheimer Disease in the Israeli Survey of Aging
Source: JMIR Aging. 2021 Apr 30;4(2):e25706. doi: 10.2196/25706 (PMC8122300; doi:10.2196/25706)
Supplement: Multimedia Appendix 1 [file aging_v4i2e25706_app1.docx]

**Multimedia Appendix 1: Knowledge on Alzheimer disease**

1. AD is a form of insanity (False)
2. If trouble with memory and confused thinking appears suddenly, it is likely due to AD (False)
3. There is currently no cure for AD (True)
4. AD could be contagious (False)
5. Genes can only partially account for the development of AD (True)
6. Difficulties in time and space orientations are symptom of AD (True)
7. AD can be diagnosed with a blood test (False)
8. Currently, the cause of AS is still unknown (True)
9. Symptoms of severe depression can be mistaken for symptoms of AD (True)
10. Most people with AD live in nursing homes (False)
